# Thirty-Second Annual Commencement of Medical Department of University of Buffalo

**Published:** 1878-03

**Authors:** 


### Editorial.

### Buffalo Medical College Commencement.

The Thirty-second Annual Commencement of the Medical Department of the University of Buffalo was held at St. James Hall on the evening of Feb. 26th. The examination by the Curators, of those recommended for the Degree was held at the College from ten A. M. till two P. M. The following is a report of their conclusion as presented to the Council of the University:—

University of Buffalo, Medical Dept.
Buffalo, N. Y., Feb. 26. 1878.

*To the Council of the University*;

The undersigned, Curators, and the members of the Faculty of the University of Buffalo, recommend the following candidates for the degree of M. D. to the Council, they having passed the requisite examination:

| | | | |
|---|---|---|---|
| J. B. Samo, | C. C. Wyckoff, | R. J. Menzie, | Jas. W. Craig, |
| W. W. Potter, | G. H. Lapham, | H. P. Hall, | Wm. Chase, |
| Edw. Storck, | S. H. Benton. | | |

THOS. F. ROCHESTER, Dean.

E. V. Stoddard, Secretary.

James Harvey Wright, Buffalo, N. Y. Wm. Ledmon Allen, Ridgeway, Ont. Chas. G. Stockton, Capeville, Va. Thos. Moore Rochester, Rochester, N. Y. John Henry Sackrider, East Randolph, N. Y. John Norcross Culbertson, Buffalo, N. Y. Ernest Wende, Millgrove, N. Y. Willis Mather Baker, Corry, Pa. Ralph DeWitt Eastman, Berkshire, N. Y. Chas. Franklin Howard, Buffalo, N. Y. Chas. Lee Van Pelt, Williamsville, N. Y. Weston Dean Bidaman, Buffalo, N. Y. Edgar Rood, Cherry Creek, N. Y. Seba Samuel Bedient, Little Valley, N. Y. DeWitt John Phillips, Oswego, N. Y. Daniel Ervin Fuller, Kenyonville, N. Y. Eugene Edward Storck, Buffalo, N. Y. John Harding, Hume, N. Y. John Frank Pratt, Mayville, N. Y. Delevan Edwin Blackman, Stockton, N. Y, Benjamin Samuel Swetland, Portland, N. Y. Richard Mott Moore, Rochester, N. Y. John Jordan Lenhart, Ellery, N. Y. Augustus Reginald Davidson, Buffalo, N. Y. Edward Moore Tompkins, Knowlesville, N.Y, Augustus Frank Miller, Lancaster, N. Y. Chauncey Abbey Rood, Cherry Creek, N.Y. James Vosburg Otto, Port Allegheney, Pa. Elijah Elliott Johnston, Stevensville, Can. James Russell Whitwood, Friendship, N. Y. George Washington Richardson, Suspension Bridge, N. Y. David Christian Eisbein, Buffalo, N. Y. Edward Hurlbert Ballou, Clarence, N. Y. Malcolm Wayne Smith, Angola, N. Y. Fred William Sweetland, Edwardsburg, Mich. Oscar Joab Stafford, Canajoharie, N. Y. Charles Arthur Ring, Buffalo, N. Y. James Christian Spiegel, Utica, N. Y. Jason Thomas Waid, Spartansburg, Pa. Oliver Hendrickson Simons, Jamestown, N. Y. Frank Wesley Sweetland, Edwardsburg, Mich. Cyrus Augustus Haskins, Benezette, Pa.

At the meeting of the Council to which the above report was presented, Prof. J. F. Miner read the following memorial:

*Mr. President and Gentlemen of the Council:*

Allow me to remind you that since our last annual meeting and during the present session, our College has lost by death two of the beloved and respected members of the faculty, Prof. Geo. Hadley, in the very height of professional distinction and usefulness, and Prof. Milton G. Potter, in the prime and vigor of early manhood. This sad and unusual occurrence deserves more than a passing notice, but it is impossible at this time to do other than give, as I may be able in a few words, expression of our deep sense of bereavement, our high appreciation of their services, of their devotion to the Medical College, to science, to truth and to mankind; a devotion so large and true as to secure for them a warm place in the hearts of their colleagues, and of every lover of medical progress and true manhood.

Prof. Hadley was one of the founders and life-long supporters of this institution, who by years of study and experience had attained eminence in his department, thus adding much to its credit and reputation. Prof. Milton G. Potter, though young in years, by his energy, earnestness and devotion to his profession, had already acquired an enviable reputation, and gave promise of great future usefulness and distinction.

The simple mention of these names brings to the members of the Faculty the kindest recollections—the deepest, truest sense of sorrow as for men who were able, earnest, faithful teachers; true, warm friends, beloved and respected in all the departments of life, associates of whom we are justly proud, who have left examples of professional and moral worth to be emulated by those of us who are

"Only waiting till the shadows
Are a little longer grown;
Only waiting till the glimmer
Of the day's last beam is flown."

The address to the graduating class was delivered by Prof. Wm. B. Wright, M. D., of the State Normal School and was an effort at once thoughtful, full of grace and power. We regret very much that lack of space prevents the publication of this charge to the young men. It will be found in full in the Buffalo papers of the 27th.

Previous to Prof. Wright's address the degrees were conferred to the graduating class by Hon. O. H. Marshall, President of the Council of the University.

THE ALUMNI MEETING.

In conjunction with the Commencement the Fourth Annual Meeting of the Association of the Alumni and Officers of the College was held. The meeting was called to order in the College Museum at ten A. M. by the retiring President, Dr. W. W. Potter, of Batavia, N. Y., who introduced the President-elect, Dr. S. H. Benton, of Corry, Pa. After a brief Inaugural Dr. Benton declared the meeting open for the transaction of business and introduced Prof. Rochester, who in a hearty manner welcomed the Alumni back to the halls of *Alma Mater*. The morning session was occupied with the transaction of routine business such as the appointment of committees, hearing of reports, etc., etc. At three P. M. the Alumni again assembled and were favored with two very interesting papers, one upon Antiseptic Surgery by Dr. C. B. Kibler of Corry, Pa., the other upon Thrombosis and Embolism by Dr. W. W. Miner. A memorial of the late Prof. Milton G. Potter was also read to the assembled Alumni, and to the members of the Erie County Medical Society, before which body it was to have been presented by appointment on the twenty-first.

The valedictory address of the President Dr. S. H. Benton, to whose remarks any synopsis which we can present would not do justice, was presented at the conclusion of the meeting. Before adjournment the following officers were elected for the ensuing year:—

President—Dr. Milan Baker, Warsaw, N. Y.
1st Vice President—Dr. D. W. Harrington, Buffalo.
2d " " Dr. C. B. Kibler, Corry, Pa.
3d " " Dr. E. C. W. O'Brien, Buffalo.
4th " " Dr. G. W. Barr, Titusville, Pa.
5th " " Dr. P. H. Flood, Elmira, N. Y.
Secretary—Dr. Wm. C. Phelps, Buffalo.

TRUSTEES.

For four years—Dr. Wm. Ring, Buffalo.
For five years—Dr. C. C. Wyckoff, Buffalo.

EXECUTIVE COMMITTEE.

Dr. A. H. Briggs, Buffalo.
Dr. W. W. Potter, Batavia, N. Y.
Dr. E. N. Brush, Buffalo.
Dean and President *ex-officio*.

At the conclusion of the Commencement Exercises at St. James Hall, Dr. Chas. Cary delivered the annual address to the Alumni. Dr. Cary's remarks, which we are also obliged to omit, were listened to with marked attention and elicited from the audience and his fellow Alumni the heartiest applause. At the conclusion of Dr. Cary's address the benediction was pronounced by Rev. D. R. Frazer, and the Faculty, Curators and Alumni, with their friends adjourned to the Ocean House, where the Annual Alumni Banquet was spread. No report of ours can give any just conception of the interesting and jolly time which now followed. Suffice it to say that "the feast of reason and flow of soul" were uninterrupted till an early morning hour. These annual gatherings are becoming a feature of Commencement time which add largely to its enjoyment, and we hope to see them long continued.

